# A system dynamics approach to stormwater harvesting under increasing drought variability

**DOI:** 10.1016/j.isci.2026.116472

**Published:** 2026-07-03

**Authors:** Oseni Taiwo Amoo, Motebang Dominic Vincent Nakin, Akinola Ikudayisi, Yusuf Lukman

**Affiliations:** 1 Centre for Global Change, Walter Sisulu University, Mthatha Campus, Eastern Cape, Mthatha, South Africa; 2 Civil Engineering Department, Walter Sisulu University, Buffalo Campus, Eastern Cape, East London, South Africa; 3 Department of Education Management, Walter Sisulu University, Mthatha, South Africa

**Keywords:** Environmental science, Geomatics

## Abstract

Increasing drought variability in South Africa’s Eastern Cape has intensified hydrological stress and exposed weaknesses in conventional water supply systems. This study combines multi-index drought assessment with a system dynamics socio-hydrological model to evaluate stormwater harvesting (SWH) in the Mthatha River catchment (2000–2020). Results show increasingly erratic rainfall, with severe droughts concentrated between 2016 and 2019. Extreme value analysis indicates that 10–20-year drought events correspond to annual rainfall of 532–512 mm, highlighting more frequent deficits. Under drought conditions, harvestable stormwater declined by 22–48%, yet optimized storage improved supply reliability by up to 35%, contributing about 0.288 Mm^3^ annually. Integrated adaptation measures significantly reduced system stress compared to single interventions. Feedback analysis shows institutional trust, economies of scale, and visibility drive adoption, while financial and infrastructure constraints limit uptake. Overall, optimized, well-supported SWH systems can enhance drought resilience and reduce municipal water dependence.

## Highlights

• Stormwater harvesting improves drought resilience in Eastern Cape systems
• System Dynamics modeling evaluated SWH under variable drought scenarios
• Optimized SWH improved supply reliability by up to 35%
• Integrated adaptation reduced dependence on conventional water systems

## Graphical abstract

**Figure:**
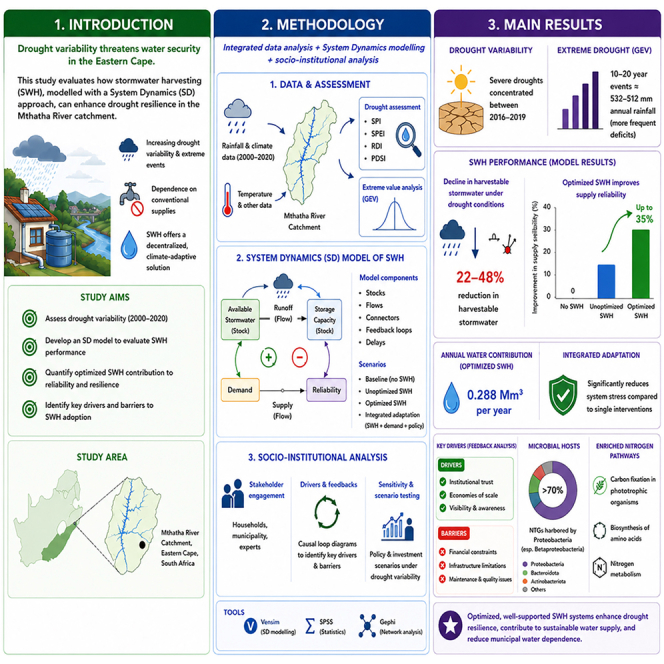

## Introduction

Drought variability has intensified globally due to climate change, disrupting rainfall patterns and increasing hydrological uncertainty.^1^^,^^2^^,^^3^ Urban areas, in particular, face escalating risks of water shortage as traditional supply systems struggle to cope with extreme climatic fluctuations. These challenges necessitate innovative, flexible, and decentralized approaches to water resource management.^4^ South Africa is classified as a water-scarce country, classified as the 30th driest in the world,^5^^,^^6^ with most provinces experiencing rising water demand driven by rapid urbanization, lifestyle changes, and economic activity.^7^

In addition, the recurrence of El Niño-related droughts, unpredictability of rainfall regimes, and persistent anthropogenic pressures, including inadequate infrastructure management and vandalism, have necessitated the exploration of alternative water-supply options.^8^ The 2015–2016 national water crisis underscored these vulnerabilities as five provinces were declared disaster areas, prompting stringent municipal water restrictions.^9^^,^^10^^,^^11^ Although drought cannot be prevented, adequate preparedness can significantly reduce its impact on ecosystems and human livelihoods. Traditional drought management approaches remain largely reactive, often initiated only after prolonged drought conditions. These approaches rely heavily on simplistic monitoring systems that inadequately characterize relationships between drought severity (DSI) and hydrological or ecological responses. Consequently, there is a need for improved drought-resilience strategies that incorporate predictive drought assessment, adaptive water resource planning, and multi-sectoral management.

Stormwater harvesting (SWH) is increasingly recognized as a climate-adaptive resource capable of supplementing conventional supplies, reducing pressure on groundwater and surface reservoirs, and improving resilience during drought periods.^12^ Accurate drought assessment provides the foundation for designing adaptive SWH systems. Conventional stormwater systems are primarily designed for drainage, not retention or reuse, limiting their role in water-supply augmentation during droughts. Developing adaptive SWH systems, therefore, represents a critical opportunity to enhance water security in regions such as Mthatha. SWH involves the collection, storage, and beneficial use of runoff as a non-traditional water resource.^13^^,^^14^ However, despite its potential to augment domestic, industrial, and agricultural supplies, its uptake in South Africa remains limited, constrained primarily by storage requirements, quality-management challenges, and spatial limitations in urban environments.^15^^,^^16^ Nevertheless, international experience demonstrates that SWH can play a key role in broader nature-based water-management strategies, complementing floodable parks, retention ponds, green infrastructure, permeable surfaces, and water-pricing reforms.^17^^,^^18^^,^^19^

Despite the potential of SWH, its performance is highly sensitive to rainfall patterns, catchment characteristics, and socio-institutional dynamics. These interactions are inherently nonlinear and shaped by feedback loops such as changing demand behavior, adaptive practices, and policy responses. Few studies integrate climatic variability, hydrological processes, and socioeconomic behavior into a unified framework capable of capturing long-term dynamics. System dynamics (SD) modeling provides a robust platform for such integration, yet its application to SWH under increasing drought variability remains limited. SD uses feedback structures and delay mechanisms to represent complex real-world phenomena through smaller, interdependent sub-systems, enabling efficient use of energy, time, and financial resources while maximizing societal value.^20^^,^^21^^,^^22^ SD models are typically composed of stocks, flows, connectors, and modifiers that describe how system components interact dynamically. Several software environments support SD modeling, including STELLA,^23^ POWERSIM,^24^ GoldSim,^25^ Simile,^26^ and Vensim.^27^ Collectively, these tools facilitate scenario testing, policy evaluation, and adaptive planning within complex systems.

The severity-duration-frequency (SDF) model developed by Smith^28^ improved the quantification of drought probabilities in South Africa, offering planners a tool to estimate the recurrence of droughts across various temporal scales. Drought indices such as the Standardized Precipitation Index (SPI), and Standardized Precipitation-Evapotranspiration Index (SPEI) are widely used for characterizing meteorological, hydrological, and agricultural drought conditions.^29^^,^^30^ SPI’s multi-timescale utility makes it particularly valuable for linking short-term and long-term drought impacts on soil moisture, streamflow, groundwater, and reservoir levels. These indices have been applied extensively in forecasting, frequency analysis, spatiotemporal drought studies, groundwater drought monitoring, and climate-impact assessments.^31^^,^^32^^,^^33^^,^^34^^,^^35^ Several of the drought variability indices have limited applicability due to data requirements, computational limitations, or interpretation challenges.^36^^,^^37^ Moreover, most indices are rarely integrated with water-resource system optimization, even though such integration can provide insights into performance under different drought scenarios.

Furthermore, most drought prediction approaches often overlook human-environment feedback. The lack of integrated frameworks linking drought dynamics to socioeconomic outcomes over time undermines the need to develop a SD model to predict drought occurrence and assess its socioeconomic consequences. This study addresses this gap by integrating feedback as an in-depth assessment of the trade-offs between water supply and demand side, incorporating the socioeconomic consequences. This study provides the first integrated assessment of SWH as a viable option to water allocation imbalance of water resources in drought scenarios using the SD framework. The study aims to.

• Analyze rainfall variability and drought trends.
• Quantify stormwater-harvesting potential under multiple drought scenarios.
• Develop a dynamic drought system model incorporating climatic, hydrological, and socioeconomic subsystems.

Hence, providing research questions on how SWH systems can be optimized to sustain water availability during drought periods?; How can SD enhance understanding of drought predictability and recurrence? What are the critical feedback loops between hydrological stress and socioeconomic vulnerability? and which policy interventions can reduce drought-induced socioeconomic risks? The research advances SDG 6 (Clean Water and Sanitation) and SDG 13 (Climate Action) by offering a systematic, climate-responsive framework for water-resource resilience in drought-vulnerable regions.

### Study area description

**Figure 1:**
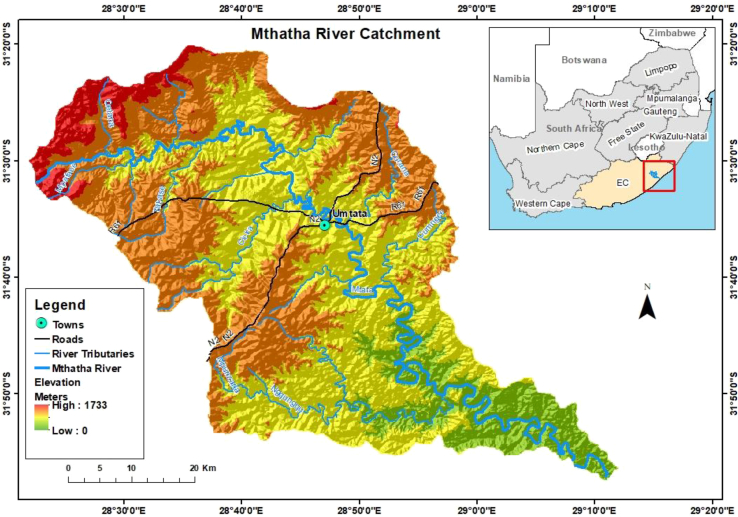
Location map of the Mthatha River Catchment

Figure 1 presents the location of the study area within the South African provincial context. Mthatha has a complex arrangement of commercial and residential settlements, including a range of dwelling types, population densities, spatial configurations, and distinct spatial characteristics evident in the city pattern.^38^ The study was conducted in the O.R. Tambo District Municipality, located between latitudes 28°31′ and longitudes 31°45′ in the Eastern Cape Province. Mthatha city is one of the region’s most rapidly urbanising cities and is characterised by complex topography comprising steep, irregular slopes and undulating terrain.^39^ The district exhibits high variability, often erratic rainfall patterns, which heavily affect streamflow characteristics and catchment-scale hydrological responses. In addition to climatic variability, drought conditions in the Eastern Cape are exacerbated by several human-induced factors, including inadequate water-resource management, vandalism of water infrastructure, and inconsistent maintenance of supply systems. Even during heavy rainfall, the hydrological response often remains insufficient to alleviate drought conditions due to poor infiltration, rapid runoff on steep slopes, and storage deficits.

### Conceptual Framework

Climate drivers → Drought indices → Severity analysis → Catchment response → Stormwater Availability → Harvesting System Design → Adaptive Measures.

## Results and discussion

The three-parameter log-logistic distribution was used for fitting the cumulative distribution function (CDF) for the SPEI because it captured the difference between precipitation and potential evapotranspiration (P – PET), which could be both positive and negative based on Vicente-Serrano et al. (2010). The relationships among SPI, PET, and SPEI were examined using Pearson’s correlation coefficients to assess the degree of association and divergence between rainfall, evapotranspiration, and DSI. The correlation coefficients were computed as:

(Equation 1) $$\rho SPI,PET=\frac{\sum_{i=1}^{n}\left({SPI}_{i}-\bar{SPI}\right)\;\left({PET}_{i}-\bar{PET}\right)}{\sqrt{\sum_{i=1}^{n}{\left({SPI}_{i}-\bar{SPI}\right)}^{2\;}\sum_{i=1}^{n}{\left({PET}_{i}-\bar{PET}\right)}^{2}}}$$

(Equation 2) $$\rho SPEI,PET=\frac{\sum_{i=1}^{n}\left({SPEI}_{i}-\bar{SPEI}\right)\;\left({PET}_{i}-\bar{PET}\right)}{\sqrt{\sum_{i=1}^{n}{\left({SPEI}_{i}-\bar{SPEI}\right)}^{2\;}\sum_{i=1}^{n}{\left({PET}_{i}-PET\right)}^{2}}}$$

where *SPI*_*i*_, *PET*_*i*_, *SPEI*_*i*_ Are the SPI, PET, and SPEI values at the i-th time point $\bar{SPI}$, $\bar{PET}$,and $\bar{SPEI}$ are the means of SPI, PET, and SPEI, respectively. Table 1 depicts the statistical summary for the autocorrelation between PET, SPI, SPEI, and DSI.

**Table 1:** Pearson’s correlation coefficient index for drought indices

Time	PET	SPI	SPEI	DSI
PET	1			
SPI	−0.0909	1		
SPEI	−0.0834	0.0926	1	
DSI	0.0554	0.0419	0.0364	1

Correlations are significant at p (alpha) = 0.05.

Strong internal PET correlations suggest coherent trends across regions. In contrast, negative PET-SPI/SPEI correlations highlight the potential for diminishing water availability, with a stronger −0.0909 and −0.0834 correlation value reinforcing projections by Ullah et al.,^40^ regarding drought intensification in response to rising PET. While PET increases, stable precipitation suggests growing risks of water stress, echoing findings by Gao et al.^41^ The combination of rising PET and steady rainfall may lead to soil moisture depletion and hydrological imbalances, posing challenges for agriculture and ecosystems. Thus, the adoption and implementation of SWH systems will help compensate for this water shortage, which can be later used in the root zone for plant use. Hence, a positive correlation between SPI and PET indicates higher precipitation, in contrast to higher PET, which suggests increased water demand for vegetation.

### Trend analysis using Mann–Kendall

Table 2 presents Sen’s slope and the Mann-Kendall test as viable tools for detecting abrupt change periods and analyzing the climatic trend patterns in the catchment. A very high positive value of “S” in the Mann-Kendall and Sen slope is an indication of an increasing trend, while a very low negative value indicates a decreasing trend.

**Table 2:** The Mann-Kendall Trend and the annual Sen’s slope results

Variables (Annual)	Kendall’s tau	S	Var(S)	*p* value (two-tailed)	Sen’s slope	Trend
Ave.Humidity	−0.571	−120	1096.667	0.000	−0.167	Decreasing
Sunshine	−0.190	−40	1096.667	0.239	0.367	Decreasing
Ave. Max. Temp.	0.114	24	1096.667	0.487	0.065	Increasing
Ave. Min. Temp.	−0.320	−67	1095.667	0.046	−0.004	Decreasing
Ave. Wind	0.114	24	1096.667	0.487	0.204	Increasing
Ave. rainfall	0.016	8	3802.667	0.910	0.586	Increasing
Ave. Evapotra	0.114	24	1096.667	0.487	0.204	Increasing
Ave. Streamflow	−0.270	−80	1096.667	0.339	0.347	Decreasing

Interpretation of Table 2 suggests statistically significant increasing trends in maximum temperature, rainfall, and evapotranspiration (*p* > 0.05), while humidity and minimum temperature exhibit decreasing tendencies. Sunshine duration and evapotranspiration show proportional variability, reflecting their dependence on solar radiation.

### The cumulative recurrence intervals of drought

The recurrence intervals of drought were calculated from an inverted generalized extreme value distribution. The GEV CDF for a value *z* is given as noted in Equation 3

(Equation 3) $$G\left(z\right)=\exp\left\{-{\left[1+\xi \left(\frac{z-\mu }{\sigma }\right)\right]}^{-1/\xi }\right\}\text{,}$$

valid for $1+\xi \frac{z-\mu }{\sigma }>0$.

Table 3 depicts the fitted parameters for location *μ*, scale *σ* > 0, and shape *ξ*.

**Table 3:** Fitted parameters for location, scale, and shape

Parameter	value
Shape (ξ)	0.6006
Location (μ)	−694.16
Scale (σ)	131.5

Hence, the GEV return levels present expected low rainfall for droughts of different recurrence intervals.

Table 4 shows the results of the expected low rainfall and the return drought period for the next 20 years.

**Table 4:** Return period and expected rainfall value

Return Period (Years)	Expected Low Rainfall (mm)
2-year drought	650.9
5-year drought	564.2
10-year drought	531.9
20-year drought	512.0

### Interpretation

A 2-year drought corresponds to annual rainfall of around 651 mm (mild drought).

A 5-year drought is around 564 mm.

A 10-year drought falls to ∼532 mm.

A 20-year drought is a severe drought with around 512 mm.

These values are consistent with the lowest historical observations (∼489–540 mm range). Hence, the need for a complementary alternate supply of adequate water requirements by plants and domestic uses, otherwise, agricultural drought may set in rapidly and can suddenly terminate the growth of the planted crop. Although agricultural water demand is the largest water requirement for the region, insufficient water to meet the demand results in agricultural drought, which is often characterized by important short-term changes in volumetric soil moisture in the root zone and a reduction in plant water requirements for healthy growth of the plant.

### The cumulative impact of drought variability

The cumulative impact of drought variability was assessed through indices such as precipitation concentration Index (PCI), Standardised Precipitation Anomaly Index (SPAI), precipitation ratio (PR), and coefficient of variability (CV) of rainfall, as illustrated in Table 5.

**Table 5:** The cumulative impact of drought variability

Year	PCI	Precipitation Ratio (PR)	Coefficient of Variation (CV)	SRAI	Ave.Rainfall (mm)(yearly)
2000	0.096	9.605	40.796	0.221	959.900
2001	0.117	11.709	66.478	−0.384	683.400
2002	0.108	10.751	56.255	0.989	653.900
2003	0.122	12.241	71.525	0.452	489.800
2004	0.111	11.066	59.808	−0.658	581.100
2005	0.104	10.374	51.682	0.345	662.500
2006	0.107	10.687	55.509	0.470	832.400
2007	0.109	10.937	58.382	0.773	529.900
2008	0.095	9.457	38.356	0.101	687.500
2009	0.095	9.495	38.992	−0.136	618.100
2010	0.127	12.707	75.671	−0.522	797.900
2011	0.103	10.266	50.300	0.651	843.600
2012	0.105	10.516	53.454	0.540	817.400
2013	0.102	10.208	49.537	0.654	712.400
2014	0.098	9.826	44.200	0.400	540.600
2015	0.114	11.379	63.145	1.610	518.100
2016	0.091	9.055	−30.727	−2.516	644.100
2017	0.209	20.926	−128.395	−1.986	616.700
2018	0.096	9.641	41.382	−1.473	633.700
2019	0.091	9.092	−31.516	−2.560	586.800
2020	0.096	9.599	40.709	0.164	688.900

The Standardized Rainfall Anomaly Index (SRAI) highlights a growing frequency of dry years, particularly severe in 2016, 2017, and 2019, indicating worsening drought conditions. Notably, years with high PCI and PR values often correspond to low rainfall and negative SRAI values, underscoring the link between erratic precipitation patterns and reduced water availability. The rest of the year in the catchment has below-normal rainfall distribution, which is less than 600 mm in a year. Overall, the data suggest that climatic variability is increasingly disrupting rainfall reliability, with implications for water resource management and agricultural planning.

Table 6 depicts the years with a negative anomaly index for very dry to arid years with severe drought experienced, while those with a positive anomaly index range from very wet to extremely wet years. The years 2015–2020 show moderately dry years till an arid year (−0.5 to −2.5), while the years 2000–2010 varied from a normal to moderate wet period. The data from 2000 to 2020 reveal a clear trend of increasing climatic variability impacting rainfall patterns, with a general decline in average annual rainfall and growing irregularity in its distribution. The PCI mostly indicates a fairly uniform distribution, except for a significant spike in 2017 (PCI = 0.209), pointing to extreme rainfall irregularity. The coefficient of variation (CV), which reflects rainfall variability, shows moderate values until 2015, after which unusually negative values appear, suggesting either data anomalies or methodological inconsistencies.

**Table 6:** Stormwater availability (cubic meters) at different dependability

Months Rainfall (mm)	*F=50%*	75%	85%
January	14.6	10.5	7.2
February	37.2	13.6	10.5
March	10.8	8.1	4.2
April	5.4	2.5	0.1
May	32.0	13.2	8.5
June	46.9	16.8	8.1
July	27.7	18	9.9
August	30.1	11.4	4.3
September	12.9	9.3	7.8
October	0.10	0	0
November	0.16	0	0
December	3.40	1.3	0
**Total**	**221.26**	**104.70**	**60.60**

The total sum of the different dependable frequencies.

### Stormwater harvesting potential across scenarios

Based on the Weibull ranking, the different percentage ranking for dependent rainfall frequency was used for rainfall convolution converted into flow. The estimated available water was calculated for the Mthatha storage at different dependability frequencies. Table 7 depicts the varied frequency of rainfall dependability for the stormwater storage capacity requirement at 50%, 75%, and 85%.

**Table 7:** The sustainability index for varied dependable flow conditions

Variables	Scenarios	Variables	60%	70%	85%
A	BaU	SI	1.0	0.25	0.36
B	Climate change (Precipitation varies in 10%)	SI	0.75	0.24	0.30
C	Irrigation improvement	SI	0.50	0.23	0.26
D	Integrated scenario	SI	0.25	0.20	0.22

**Figure 2:**
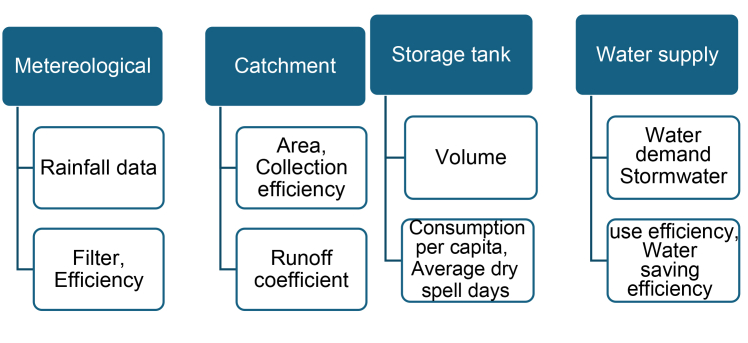
SWH components and design parameters

The Stormwater yield simulations show that storage designed for 85% dependable rainfall provides the most reliable performance under dry conditions. This was followed by 75% dependable flow that resulted in 104.7 mm, followed by 50% dependability that required a large storage capacity of 221.26 mm. Three drought scenarios were simulated: baseline (normal variability), moderate drought, and severe drought. Harvestable volume calculated as: Q = C × I × A, where: C = runoff coefficient, I = rainfall depth, and A = catchment/roof area. The harvestable stormwater declined by 22–48% under moderate to severe drought scenarios, while the storage efficiency depended strongly on system sizing and rainfall distribution, except that optimized tank volumes improved up to 35% reliability for severe drought conditions. Using the integrated datasets to give the SD model numerical realism. The simulated SD result for the yearly yield for an adequate supply for domestic use, supporting water availability of approximately 0.288 Mm^3^ annually. However, SWH effectiveness depends on appropriate storage sizing, infrastructure design, land-use planning, and behavioral adaptation. Incorporating optimization analysis into harvesting system design offers a novel contribution to current literature, highlighting trade-offs between storage capacity, reliability, and cost efficiency as depict in Figure 2. The optimized SWH system improves water-use efficiency and reduces municipal water dependency, providing measurable economic benefits and enhancing drought resilience.

### System dynamics CLD results discussion

**Figure 3:**
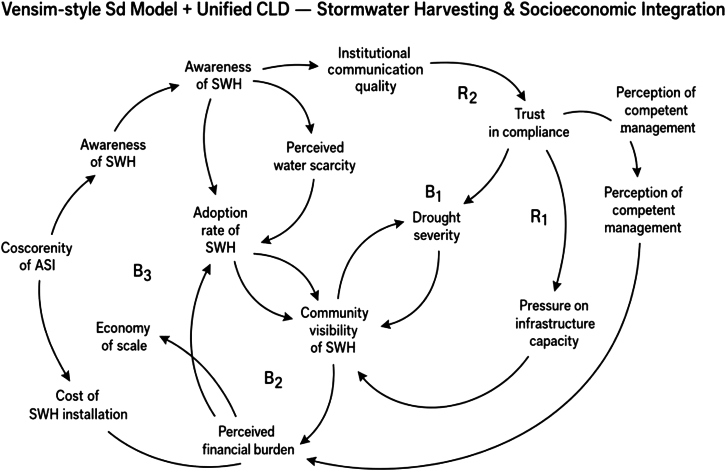
Causal loop diagram for SHW and socioeconomic integration

The resultant results for the conceptualized CLD framework are as depicted in Figure 3. The connector groups are listed, and the loop names with their short descriptions. Hence, as household SWH coverage increases, the visibility of SWH systems within the community rises, thereby enhancing public awareness and strengthening households’ willingness to adopt SWH. Also, an increase in perceived benefits further boosts willingness to adopt, while higher SWH costs raise the perceived financial burden, reducing household adoption intentions. The connector-Household_SWH_Coverage -> Economies_of_Scale (+) was interpreted as household SWH coverage rises, overall municipal water demand decreases through greater substitution with harvested stormwater, thereby reducing storage outflow. While connector designated as Economies_of_Scale -> Cost_of_SWH (−), Municipal_Infrastructure_Capacity -> Adoption_Effectiveness (+), Household_SWH_Coverage -> Demand_reduction_via_SWH (+) and Demand_reduction_via_SWH -> Storage_Outflow (−) were interpreted as the higher DSI increases perceived water scarcity, which improves compliance with water-use restrictions and drives down per-capita water demand, liekewise, trust in municipal institutions strengthens both compliance with restrictions and willingness to adopt SWH systems, for as SWH adoption grows, pressure on municipal infrastructure increases, which, unless offset by targeted investment, reduces infrastructure capacity over time. Overall, investments in capacity expansion counteract this decline by enhancing municipal infrastructure capabilities.

### Causal loop dynamics of household SWH adoption

The Vensim loops diagram reinforces and balances the contextual model stocks, flows, auxiliaries, and equations. The **Loops (labelled-R1)—Stormwater Harvesting Adoption (reinforcing)** Household_SWH_Coverage (+) - > Community_Visibility (+) - > Awareness (+) - > Willingness_to_Adopt (+) - > SWH_Adoption (+) - > Household_SWH_Coverage.

**B2—Economic Constraint (balancing)** Household_SWH_Coverage (+) - > Economies_of_Scale (+) - > Cost_of_SWH (−) - > Perceived_Financial_Burden (−) - > Willingness_to_Adopt (+) - > SWH_Adoption (+) - > Household_SWH_Coverage (Note: this loop can be reinforcing or balancing depending on parameterization; treat as balancing when high costs suppress adoption.).

**B1—Water Demand Response (balancing)** Drought_Severity_Index (+) - > Perceived_Water_Scarcity (+) - > Compliance_with_Restrictions (+) - > Demand_per_Capita (−) - > Storage_Outflow (−) - > Water_Storage (+) - > Drought_Severity_Index (−).

**R2—Institutional Trust & Compliance (reinforcing)** Institutional_Communication_Quality (+) - > Institutional_Trust (+) - > Compliance_with_Restrictions (+) - > Effectiveness_of_Restrictions (+) - > Perception_of_Competent_Management (+) - > Institutional_Trust.

**B3—Infrastructure Capacity Pressure (balancing)** SWH_Adoption (+) - > Pressure_on_Infrastructure (+) - > Municipal_Infrastructure_Capacity_change (−) - > Adoption_Effectiveness (−) - > SWH_Adoption.

**Figure 4:**
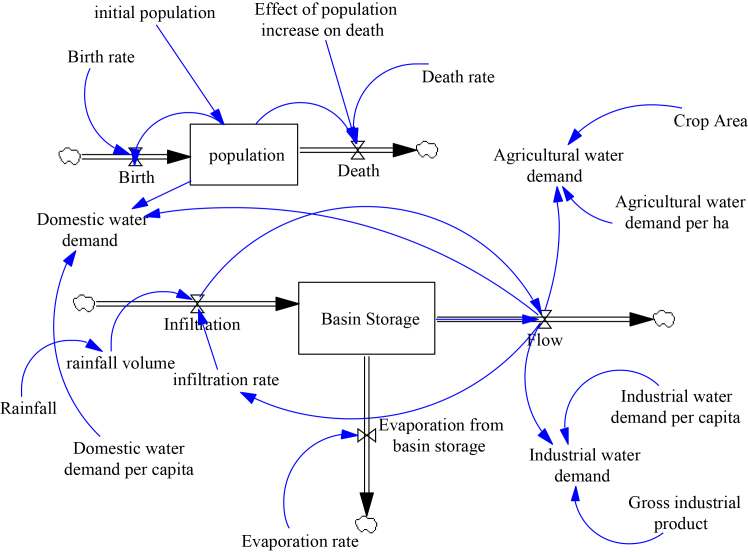
The SFD simulates water storage, flow, and water demand variables

The SD analysis reveals a set of interlinked reinforcing and balancing feedback that shapes the behavior of household SWH adoption. Increases in household SWH coverage enhance community visibility of the technology, which elevates public awareness and strengthens willingness to adopt, forming a self-reinforcing visibility-awareness-adoption loop. Perceived benefits further amplify this adoption tendency, while expanding coverage generates economies of scale that progressively reduce system costs and lower financial barriers, creating an additional reinforcing pathway that accelerates uptake. However, these adoption drivers are moderated by a balancing financial burden loop in which higher system costs increase the perceived financial burden, reducing willingness to adopt and dampening growth in coverage. In addition, improvements in municipal infrastructure capacity contribute positively to adoption effectiveness, enabling better integration of SWH systems and supporting their sustained use. Expanding household coverage also reduces municipal water demand through increased substitution with harvested stormwater, which subsequently lowers storage outflow and stabilizes system pressures. External climatic stressors further shape behavioral responses: greater DSI heightens perceived water scarcity, which enhances compliance with water-use restrictions and reduces per-capita demand. Institutional trust plays a crucial mediating role by increasing both compliance with restrictions and willingness to adopt SWH, reinforcing the behavioral shifts needed to sustain water-saving practices. As adoption scales up, increasing pressure is placed on existing municipal infrastructure, which—if not counteracted—reduces overall system capacity and constrains the effectiveness of further adoption. Targeted investments in capacity expansion counterbalance these pressures by strengthening infrastructure performance, enabling continued growth in SWH adoption. Together, these interacting, reinforcing, and balancing loops illustrate how social perception, economic drivers, institutional factors, infrastructure capacity, and climatic stress collectively shape the dynamic trajectory of household SWH adoption (Figure 4).

### System dynamics calibration and validation result discussion

Model calibration involved adjusting parameters to reproduce historical water demand and drought patterns. Qualitative validation was performed through stakeholder feedback and structural verification, ensuring alignment between the model logic and interview insights. Quantitative validation included behavior reproduction tests, extreme-condition tests, and sensitivity analysis. This integrated methodology enabled the SD model to represent both the physical water system and the social behaviors shaping resilience and SWH uptake. The optimized SWH potential across the scenarios was tested for the sustainability index (SI) as defined by Xu et al.^42^ (2002) using Equation 4.

(Equation 4) $$SI=\left\{ \begin{array}{c} \frac{\left(S-D\right)}{S}\\ 0 \end{array}\right.\;\begin{array}{l} S>D\\ S\leq D \end{array}$$

where S is the available water supply, and D is the water demand. The SI combines the ratio of aggregated possible water demand relative to the corresponding supply at the same time, in validating the medium and long-term water fulfillment from both sides (supply and demand) at varied dependability flow conditions (Table 6).

**Figure 5:**
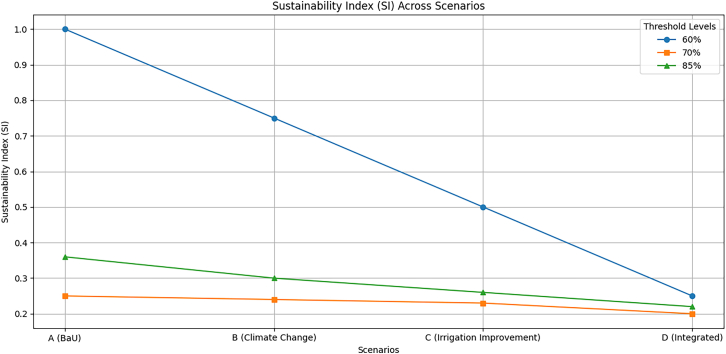
The Integrated Scenarios’ Sustainability Index

SI values smaller than 0.25 correspond to low or no stress on water supply, which implies that water demand is less than or equal to 80% of the potential water supply, whereas those greater than 0.25 reflect vulnerable conditions, i.e., water demand is greater than 80% of the potential water supply. Values of zero indicate an unsustainable water supply, i.e., water demand already equals or exceeds all available local water resources. The result of the integrated scenarios (B + C) (Table 7) of the SD sustainability index at varied dependability conditions in relation to the basin storage basin not only predicts the likely hydrological dynamics variation at a given time but also reveals the need for increased storage capability to cater to the growing population. Figure 5 discusses the integrated scenario sustainability (SI) under alternative management and climate scenarios. across 60%, 70%, and 85% performance thresholds.

Using the threshold-specific insights- 60% threshold SI declines sharply from 1.0 (BaU) to 0.25 (integrated), highlighting strong gains when adaptation and management measures are combined. The 70% threshold depicts differences between scenarios as narrow, suggesting moderate sustainability sensitivity once higher performance requirements are imposed, while 85% threshold shows the Integrated scenario performs best (lowest SI = 0.22), indicating superior robustness under stringent sustainability conditions. Hence, the Integrated scenario consistently outperforms single-intervention scenarios, demonstrating that combined climate adaptation and irrigation improvements yield the most sustainable outcomes across all performance thresholds.

### System dynamics simulation outputs for water demand and supply

Water scarcity intensified nonlinearly during prolonged droughts. Also, there will be an increased SWH adoption under scarcity-driven reinforcing loops, while the socioeconomic vulnerability rose drastically under severe drought without intervention. Hence, the key leverage points from the identified feedback mechanisms are increasing storage capacity, incentivizing demand reduction, accelerating SWH adoption, and implementing tiered tariff structures. Likewise, there is a need for an increased storage basin over time. As the population increases, the various water demand sub-sectors will continue to exceed the available water in the storage basin. Thus, necessitating measures for regulating water use, which may include creating awareness to avoid water wastage in its various uses, crop diversification and precision water use in farming, the use of metering and pricing for water auditing.

### Implications for socio-economic oscillation

**Figure 6:**
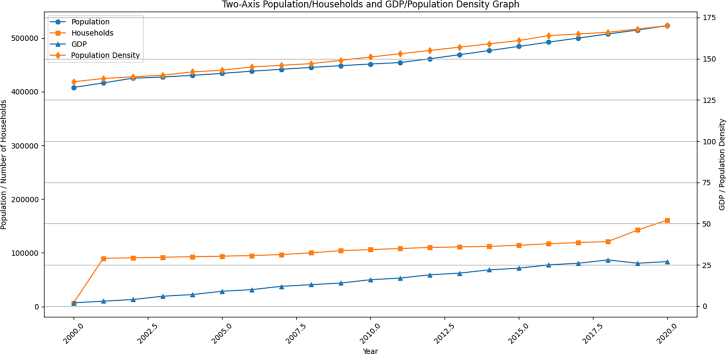
A combined demography and economic indicator graph for MRC

The aggregated demographic and economic indicator oscillation strata over the year are depicted in Figure 6.

Figure 6 presents the temporal evolution of key demographic and economic indicators between 2000 and 2020. As shown in Figure 6, the total population increased steadily over the study period, rising from approximately 408,000 in 2000 to over 522,000 by 2020, reflecting sustained demographic growth. Population density followed a similar upward trajectory, increasing consistently across the period, indicating intensifying settlement pressure. The number of households also exhibited a general upward trend until approximately 2016, after which a pronounced decline was observed, suggesting a discontinuity in household data that coincides with changes in measurement or classification rather than a reversal of demographic growth. Economic dynamics displayed in the right axis (GDP & population density) reveal a more heterogeneous pattern. Gross domestic product (GDP) increased markedly over the study period, particularly after 2010, indicating expanding economic activity. The economically active population remained relatively stable in the early years but showed a gradual increase from the mid-2010s onward. Employment levels rose substantially over time, increasing more than 4-fold between 2000 and 2020, although short-term fluctuations are evident, particularly around the late 2000s and early 2010s. Overall, the combined trends suggest that demographic expansion was accompanied by growing economic activity and employment, albeit with differing rates of change across indicators. When there is a steady growth in population and household numbers, it has implications for the volume of storage, river flows, groundwater, and sectoral demands. Persistent drought conditions decrease the amount of water flowing in streams and increase competition for water supplies, highlighting the ongoing problem of water vulnerability in these areas- Figure 6.

**Figure 7:**
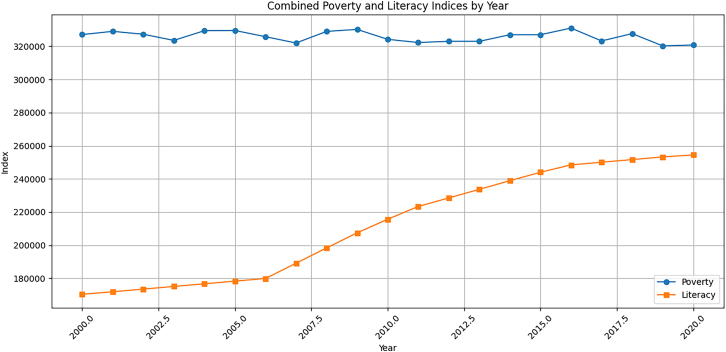
Combined poverty and literacy opportunity indices

A cursory look at Figure 7 reveals that the poverty and literacy indices depicting the Mthatha catchment have maintained a steady poverty rate over the study period, with a similar trend observed in literacy. The combined poverty and literacy opportunity indices illustrate divergent long-term trends in welfare and human capital development between 2000 and 2020. The Poverty Index has been used as the best way to measure the region’s standard of living (DWAF 2004; Brown et al. 2015). Even though the economic activities among the populace were observed to be twice the population, this tends to impact access to water and employment opportunities. The figure shows an average economic growth rate of 3.8% for the period, with agricultural workers accounting for 78.3% of the province’s total. Meanwhile, the catchment poverty index assumes a fairly constant (300,000) literacy rate per year; growth tends to increase arithmetic as per capita income distribution in the basin (DWAF 2004). The figure clearly shows that many of its citizens did not have access to a basic water supply. This number is no match for the growing demand in the O.R. Tampo municipality and the Eastern Cape Province in particular (DWA 2009). Thus, the adoption of the SWH system will serve as an alternate storage coping mechanism for the area.

The Literacy Index increases steadily and substantially, reaching nearly 150 by 2020, indicating strong cumulative gains in human capital over the study period, with the poverty index remaining relatively flat and slightly declining, fluctuating close to the baseline value with no sustained upward improvement. The widening gap between the two indices over time visually captures a growing “opportunity paradox” showing improvements in literacy (capability expansion) are not matched by proportional reductions in poverty (welfare outcomes). Further examination of social indicators reveals that improvements in economic and labor-market conditions were not uniformly mirrored across welfare outcomes.

Despite sustained population growth and rising employment (Figures 6 and 7), poverty levels remained persistently high throughout the study period, fluctuating within a relatively narrow range and exhibiting only modest reductions toward later years. This suggests that employment growth did not translate proportionally into poverty alleviation. In contrast, access to improved water services increased steadily over time, rising from approximately 48,000 beneficiaries in 2000 to about 78,000 by 2020, indicating progressive expansion of basic service delivery alongside demographic and economic growth. Literacy levels also showed a consistent upward trend, increasing substantially over the study period, which aligns with long-term investments in human capital rather than short-term economic cycles. Collectively, these patterns indicate that while economic expansion and employment growth coincided with gradual improvements in service access and literacy, poverty reduction lagged, highlighting uneven social outcomes amid sustained demographic and economic change.

### Adaptive strategies and management implications

Recognizing both the use and non-use values of SWH supports more equitable and efficient distribution policies. The multiple-reuse nature of stormwater aligns strongly with these principles, offering a superior alternative to single-use stormwater drainage systems. Current drought-response measures in the region remain predominantly reactive, focused on hydrological and meteorological indicators without addressing the systemic drivers of vulnerability. This study introduces the use of optimization analysis to assess SWH performance under varying drought severities, identify adaptive storage-sizing strategies, and water conservation measures for adaptation. Such analytical approaches offer new insights into water-security trade-offs and provide a scientifically grounded basis for resilient catchment management.

This research demonstrates the potential of SWH systems as viable and sustainable alternatives to augment water supply during drought conditions. The study provides policymakers and urban planners with evidence on the economic and operational benefits of multi-purpose stormwater utilization, highlighting its role in increasing water-use efficiency and reducing reliance on conventional municipal supplies. As water scarcity intensifies due to rising demand and climate-driven reductions in supply, transparent and economically efficient water-allocation principles become increasingly critical.

This study presents an integrative SD framework for evaluating SWH under escalating drought variability. The study demonstrates that drought variability in the Mthatha River catchment is intensifying, with rising evapotranspiration and temperature, declining streamflow and humidity, and increasingly erratic rainfall patterns. Severe drought anomalies between 2016 and 2019, together with extreme value projections indicating 10–20-year return-period droughts at approximately 532–512 mm annual rainfall, confirm escalating hydrological risk. These quantified trends highlight that future water planning cannot rely solely on conventional surface storage systems.

SWH presents a measurable adaptation pathway. Although harvestable volumes decline by 22–48% under moderate to severe drought, optimization of storage sizing improves supply reliability by up to 35% and can augment approximately 0.288 Mm^3^ of domestic water annually. Sustainability index simulations further show that integrated adaptation strategies, combining climate-responsive planning and irrigation efficiency, reduce system stress from SI = 1.0 under business-as-usual conditions to SI = 0.22 under stringent (85%) performance thresholds, outperforming isolated interventions.

The SD results identify clear policy leverage points: (1) strategic expansion of decentralized storage capacity; (2) financial incentives and tiered tariff structures to accelerate SWH adoption, (3) strengthening institutional communication to enhance compliance and trust, and (4) integration of SWH into municipal infrastructure and spatial planning frameworks. Without coordinated intervention, demographic growth and rising sectoral demand will continue to outpace basin storage capacity. Practically, municipalities should adopt optimized SWH design standards linked to drought recurrence intervals, mainstream SWH within building regulations, and combine infrastructure investment with behavioral and governance reforms. The integrated SD framework developed in this study provides a transferable decision-support tool for climate-adaptive water planning in drought-prone urban catchments.

### Limitations of the study

The proposed SD-based framework provides a scalable and transferable tool for climate-adaptive water planning in drought-prone regions. However, subject to several methodological, data, and decision-support limitations, decision makers and water resource managers should interpret our results in line with these limitations. Many feasible solutions appear at the system level but are impractical locally. These limitations are related to abstraction, uncertainty, and decision relevance. Its reliance on aggregated system representations oversimplifies spatial, sectoral, and infrastructural heterogeneity, while hydrological and engineering processes are treated in a stylized manner rather than a physically explicit form. Also, among the study limitations were discrepancies in historical data records and assumptions regarding spatial variability in stormwater generation, which were not fully represented in this study. Understanding hydro-climatic relationships within the catchment was restricted by the available information on water balance components. Many conclusions drawn in this study were validated through the behavioral observation of recent drought events. The model’s behavior is highly sensitive to uncertain parameters and scenario assumptions, particularly under extreme or unprecedented drought conditions, and calibration and validation are often weak due to limited historical data. SD’s coarse temporal resolution favors long-term policy exploration but limits its usefulness for short-term drought emergency operations. Human behavior, institutional capacity, and governance dynamics are also simplified, potentially overstating the feasibility and timing of augmentation interventions. Consequently, SD models are better suited for exploration than predictive decision-making and require integration with hydrological, optimization, or agent-based models to support robust drought management.

## Resource availability

### Lead contact

Oseni Taiwo Amoo; ejire36@gmail.com.

Requests for further information and resources should be directed to and will be fulfilled by the lead contact, Oseni T. Amoo (ejire36@gmail.com).

### Materials availability

All materials used have been disclosed to the public and are also available from the lead contact without restriction.

### Data and code availability

Any additional information required to reanalyze the data reported in this paper is available from the lead contact upon request. Also, all data and code used have been disclosed to the public.

## Acknowledgments

The authors would like to acknowledge the National Research Foundation (NRF) assistance through the Centre for Global Change at Walter Sisulu University, Mthatha. Also, we are grateful to the staff and student colleagues at the center who helped with the data collection. We are indeed grateful for the constructive comments and suggestions from an anonymous reviewer, which have improved the presentation of the paper. Opinions expressed and conclusions arrived at are those of the authors.

## Author contributions

Conceptualization, O.T.A. and M.D.V.N.; methodology, O.T.A.; investigation, O.T.A. and A.I.; writing – original draft, O.T.A.; writing—review and editing, Y.L. and M.D.V.N.; funding acquisition, M.D.V.N.

## Declaration of interests

The authors declare no competing interests.

## Declaration of generative AI and AI-assisted technologies in the writing process

During the preparation of this work, the author(s) used [ChatGPT tool] in order to improve the coherence, fluency and reduce redundancy. After using this service, the author(s) reviewed and edited the content as needed and take(s) full responsibility for the content of the publication.

## STAR★Methods

### Key resources table

**Table:** 

REAGENT or RESOURCE	SOURCE	IDENTIFIER
**Software and algorithms**		

System dynamics in the VENSIM environment	The official Vensim documentation, the textbook *Business Dynamics* by John Sterman, and academic tutorials focusing on modeling, debugging, and policy simulation	Winz, Brierley and Trowsdale^43^; Sapiri, H., Zulkepli, J., Ahmad, N., Abidin, N.Z. and Hawari, N.N.,^27^ https://vensim.com/resources/

**Other**		

XLSTART statistical software, Microsoft Excel	Microsoft	N/A

### Experimental model and study participant details

Omitted as our study does not involve biological models.

### Method details

The methodological framework included assessing the likelihood and severity classes of drought with the associated socioeconomic consequences. Socioeconomic interview data were used to build and justify the model structure, while quantitative datasets were used to parameterise, calibrate, and validate the model. Together, they created a mixed-methods system dynamics model that captures both the physical water system and the social behaviours influencing drought resilience and stormwater harvesting. Trend analysis was performed using the non-parametric Mann–Kendall (MK) test combined with Sen’s slope estimator to detect monotonic trends and quantify their magnitude. Three drought indices were employed to characterise drought severity within the catchment between 2000 and 2020. This includes PET, SPI, and SPEI. These indices were used to provide a multi-dimensional understanding of drought conditions, integrating atmospheric, hydrological, and catchment-flow components.

The drought severity indicators were calculated from measured data. The SPEI is an extension of the SPI, which considers precipitation and potential evapotranspiration (PET). It quantifies droughts by incorporating the water balance (precipitation minus PET) and is calculated as:

$$SPEI=\frac{\left(P-PET\right)-\mu P-PET}{\sigma P-PET}$$

Where: *P* = Precipitation (mm), *PET* = Potential Evapotranspiration (mm), *μP − PET* = Mean of the difference (precipitation minus PET) over a reference period, *σP − PET* = Standard Deviation of the difference (precipitation minus PET) over the reference period.

The Standardized Precipitation Index (SPI) is a widely used indicator to quantify precipitation anomalies and assess drought conditions. It measures the deviation of precipitation from its long-term average over different timescales (1 month, 3 months, 6 months, etc.). This indicator was calculated:

$$SPI=\frac{P-\mu P}{\sigma P}$$

Where: *P* = Observed Precipitation for the specific period, μP = Mean precipitation for the reference period (Usually long term, like 30 years), = Standard Deviation of Precipitation for the reference Period.

#### Potential Evapotranspiration (PET)

The Thornthwaite method estimates PET based on mean temperature and the annual heat index. It is most suitable for regions where only temperature data is available.

$$PET=16{\left(\frac{T_{mean}}{5}\right)}^{1.514}\times \frac{I}{I_{sum}}$$

$$I=\sum_{n=1}^{12}{\left(\frac{T_{mean}}{5}\right)}^{1.514}$$

Where: *T*_*mean*_ = Mean temperature(°C), *I* = Heat index for the month, *I_sum_* = Sum of heat index over the 12 months. In the other hand, the Hargreaves method estimates PET based on temperature and temperature range (maximum and minimum). The formula is:

$$PET=0.0023\times \left(T_{mean}+17.8\right)\times {\left(T_{\max}-T_{\min}\right)}^{0.5}\times R_{a}$$

Where: *T*_*mean*_ = Mean temperature (°C), *T*_*max*_ = Maximum Temperature (°C), *T*_*min*_ = Minimum Temperature (°C), R_a_ = Extra-terrestrial radiation, calculated based on latitude and day of the year. Against all, the Makkink method is based on both temperature and precipitation. This index was calculated:

$$T=0.19\times \left(\frac{T}{T_{0}}\right)\times \left(\frac{P}{P_{0}}\right)$$

Where; T = Mean temperature (°C), T_0_ = Reference temperature (150C), *P* = Precipitation (mm) *P_0_* = Reference Precipitation value (60 mm).

#### System Dynamics - Socio-Hydrological Feedback

The study employs a System Dynamics (SD) modelling approach implemented in Vensim to capture the nonlinear feedback between hydro-climatic variability, water demand, and stormwater harvesting interventions. A stock–flow (Figures 2 and 3) structure was developed to represent water storage dynamics, incorporating runoff generation, harvesting efficiency, and consumption processes. The model was parameterized using historical data and validated through behavioral reproduction and sensitivity analysis. Scenario simulations were conducted to evaluate the resilience-enhancing potential of stormwater harvesting under varying drought intensities, with outputs informing the system-level dynamics illustrated in the graphical abstract.

The adaptive coping mechanisms for long-term water sustainability were simulated in the Vensim environment. Using the necessary parameters from document S1 method S1 and S2 and their utilization in the theoretically conceptualized Causal loop diagram for the system dynamic model framework, as depicted in Figure 2.

The CLD indicates how a complex web of socio-economic and environmental challenges in a catchment could be managed sustainably. The stock flow diagram (SFD) relates the various sub-system feedback to the socio-hydrological mass balance of the catchment (Figure 3). The linkages between drought severity, water shortages, household vulnerability, and socio-economic impact require adaptation strategies.

Figure 3 illustrates water demand scenarios where drought-adjusted rainfall and evaporation data were used to assess the system yield, efficiency, and reliability under dry conditions. In deriving Stormwater availability and designing a harvesting system, Figure 4 depicts the SWH component and design parameters.

Also, Microsoft Excel was used to store the parameter values for each of the riparian water demands,^44^^,^^45^^,^^46^^,^^47^ along with the necessary GIS shapefile from DWS.^48^ The riparian water demand includes data analysis for livestock, domestic, industrial, and environmental/ecological water demand. Each of the sectoral water demands has been calculated based on the Hussain, Thrikawala and Barker^49^ method, while the livestock water demand has been formulated after Wu et al.^50^ The study area has farmland with an area of 6,373.9 hectares with four different major crops, namely maize, sugar cane, lucerne, and pecan nuts, planted on 361 fields.^46^ Commercial afforestation planting is also significant in the area.

Water Demand for Domestic use can be calculated as in Equation 5

(Equation 5) $$Q=\left(\text{Wc}\times N\times S_{d}\right)\times 365$$

Where: $Wc=water\;consumed\;per\;person\;\left(taken\;as\;135\frac{m^{3}}{d}\right)$; *N*=*Population* and an adjusted seasonal difference in demand *S*_*d*_ (%). This fractional percentage representing the seasonal difference is the monthly annual resident demand.

The agricultural water demand is taken as the irrigation water used. The mathematical equation for the minimum total irrigation water required is as presented in Equation 6.

Minimise

(Equation 6) $$TIRWU_{vol}=\sum_{i=1}^{n}\left(\left(CWR_{i}\times S_{i}\right)/e_{i}\times A_{i}\right)$$

where *TIRWU*_*vol*_ is the total irrigation water use in m^3^ and CWRi is the total annual estimated gross crop water requirement under irrigation, in m^3^, for crop i. Si is the monthly fraction of the annual demand for crop i (%) and ei is the efficiency of water use by crop i (%). The crop water requirement (CWR) can be estimated broadly using the inductive method based on standard crop deltas.^51^

Livestock’s daily water requirement varies among animal species.^44^ Thus, Livestock Water Demand is calculated as in Equation 7

(Equation 7) $$TCWD=\left(WB*WDPWB+CB*WDPCB\right)*365*10^{-3}$$

Where WB and CB represent warm-blooded livestock and cold-blooded livestock, respectively, in m3/day units, WDPWB and WDPCB signify Water Demand per warm-blooded livestock and cold-blooded livestock, respectively, in m^3^/day.

The industrial water demand can be estimated as the Gross Industrial Product multiplied by the average water demand per unit of Gross Industrial Product/per capita $\left(\frac{m3}{year}\right)$. Equation 8 connotes its calculation.

(Equation 8) TIWD=WDIP∗GIP∗365

Where: WDIP is the Water Demand per Industrial Product, and GIP is the Gross Industrial Product (m^3^). SAPPI-SAICCOR’s industry, which exists at the mouth of the Mthatha River and irrigation farming, is the largest water user in the area.

### Quantification and statistical analysis

#### Exact stock–flow model (vensim structure)

**Core Stock Equation**.

Water Storage(t) = Water Storage(t - dt) + (Runoff + Harvesting Inflow − Demand − Evaporation) ∗ dt.

**Flow Equations**

1. Runoff Generation

Runoff = Rainfall × Runoff Coefficient

2. Stormwater Harvesting (KEY INTERVENTION)

Harvesting Inflow = Runoff × Capture Efficiency × Adoption Factor

3. Water Demand

Demand = Population × Per Capita Use × Demand Adjustment

4. Evaporation- Loss

Evaporation = Water Storage × Evaporation Rate

5. Feedback Loops

**Reinforcing Loop (R1): SWH Adoption**

Higher storage → improved reliability → increased trust → higher adoption → more harvesting

Adoption Factor → Harvesting → Storage → Reliability → Adoption Factor

**Balancing Loop (B1): Water Stress**

Higher demand → storage depletion → increased stress → reduced availability

**Climate Loop (B2): Drought Shock**

Drought ↓ rainfall → ↓ runoff → ↓ storage → ↑ stress index

**Key Output Indicators**

**Stress Index**

Stress Index = Demand/Water Storage

**Resilience Index**

Resilience = Water Storage/Demand
