# Supplementary material for: A system dynamics approach to stormwater harvesting under increasing drought variability
Source: iScience. 2026 Jul 3;29(7):116472. doi: 10.1016/j.isci.2026.116472 (PMC13378009; doi:10.1016/j.isci.2026.116472)
Supplement: Document S1. Methods S1 and S2 [file mmc1.pdf]

iScience, Volume *29*

Supplemental information

A system dynamics approach to stormwater harvesting under increasing drought variability

Oseni Taiwo Amoo, Motebang Dominic Vincent Nakin, Akinola Ikudayisi, and Yusuf Lukman

Methods S1: Parameter values used for each of the riparian water demands

Variables	Parameters	Source
Mthatha medium variant population (2001-2020)	3,609,259	(Statistics SA 2020)[48]
Gross industrial product per annum	101.86	(DWAF 2004)[46]
Domestic water demand per capita	135m^3	(DWA 2009)-[52]
Industrial water demand per capita	44Mm^3/annum	(Moseki, Tlou, and Ruiters 2010)-[53]
Water Irrigation demand per crop per hectare	475m^3	(DWAF 2014) [46]
Water demand per cold-blooded animal (WDCDA)	10 L/(capita*day)	(DWAF 2014)
Water demand per warm-blooded animal	45 L/(capita*day)	
(WDWDA)		(DWAF 2014)
Seasonal difference in demand (Ss and Si)	5%	

Method S2: Crop water requirement and planting areas for the crops

S/N	Crop	Crop water requirement (mm)
1	Maize	620
2	Sugarcane	810
3	Lucerne	1800
4	Pecan nuts	1920
